# A “Lightweight” Support Circle: How Urban Chinese Older Adults Reconfigure Online Peripheral Ties Through Low-Cost Digital Practices

**DOI:** 10.3390/bs16081430

**Published:** 2026-08-19

**Authors:** Huijun Wu, Yan Ge

**Affiliations:** School of Media and Communication, Shanghai Jiao Tong University, 800 Dongchuan Road, Minhang District, Shanghai 200240, China

**Keywords:** old adults, social support, well-being, digital peripheral ties, social media, weak ties, socioemotional selectivity theory, social convoy model, WeChat, qualitative research

## Abstract

Although peripheral ties are essential for social integration, traditional aging theories suggest that they naturally atrophy as older adults withdraw from physical social settings and prioritize intimate relationships. Digital technologies, however, offer new affordances that enable older adults to actively sustain such ties. Through six online focus groups with 32 older Chinese WeChat users (aged 55–74), this study examines how older adults manage their peripheral networks in the digital environment. Three findings emerge. First, older adults maintain a distinct typology of digital peripheral ties—Former Acquaintances, Present-day Acquaintances, and “Net Friends.” Second, they sustain these ties through strategically optimized observational engagement, tracking their networks with minimal interpersonal friction. Third, the benefits of these ties—practical information, identity maintenance, and direct emotional companionship—converge into a lightweight yet meaningful support circle that complements the strong-tie core. By uncovering these practices, this study shows that older adults are active curators of their digital social networks, providing empirical insight into the reconfiguration of late-life social convoys and design implications for age-friendly platforms.

## 1. Introduction

Social media has become a defining feature of contemporary social life. Its technological affordances, such as asynchronous messaging, persistent content, and ambient awareness, allow people to stay connected across distance and time in ways that were not previously possible (Bayer et al., 2020; Treem & Leonardi, 2013; Wellman et al., 2001). Even seemingly minimal digital actions, such as tapping “like” on a post, scrolling through friends’ feed updates, or quietly following group conversations, can generate meaningful feelings of connection (Anderl & Utz, 2025; Brewer et al., 2021; Levordashka & Utz, 2016; P. L. Liu & Yeo, 2024; Stsiampkouskaya et al., 2023). Notably, these functional characteristics have proven especially consequential for people’s interpersonal networks, and in particular for peripheral ties (Ali et al., 2023; Ellison et al., 2007; D. Liu et al., 2016), which have long been recognized as key mechanisms for accessing non-redundant information, bridging heterogeneous communities, and sustaining social integration (Granovetter, 1973). The demonstrated capacity of social media to expand and maintain these ties naturally draws research attention to one demographic group for whom peripheral ties have traditionally been most fragile: older adults.

Two compounding life-course effects explain why peripheral ties are particularly vulnerable in later life. Subjectively, according to Socioemotional Selectivity Theory (SST), as perceived future time diminishes, older adults come to prioritize emotionally rewarding intimate interactions, and peripheral connections gradually recede as a consequence (Carstensen, 1992). Objectively, the social convoy model supplies a complementary lens by foregrounding the role of life-focus transitions. As older adults withdraw from work and other production-based settings, the routine co-presence and shared role contexts that once organically sustained outer-convoy ties dissolve (Kahn & Antonucci, 1980). Under the combined weight of motivational selectivity and contextual dissolution, older adults’ peripheral ties appear to be locked into a well-documented structural predicament.

Yet this predicament, at least in terms of social opportunity and context, is beginning to erode. Even though their usage rates still lag behind younger cohorts, older adults around the world are adopting social media at an accelerating pace (Cotten et al., 2022). In China, this trend is especially pronounced. By December 2024, internet penetration among those aged 60 and above had reached approximately 52.5%, and this group constituted 14.1% of the country’s 1.1 billion internet users (CNNIC, 2025). As social media platforms such as WeChat become deeply embedded in daily life, they may be opening up new forms of interpersonal interaction, new social contexts, and new opportunities for older adults.

Against this backdrop, a compelling question emerges: Could older adults’ peripheral ties be revitalized with the support of social media? If so, what do these online peripheral ties look like? How are they maintained through the interaction between older adults and digital platforms? And what benefits do older adults derive from them?

Despite growing scholarly interest in older adults’ social media use, the predicament and potential digital re-emergence of older adults’ peripheral ties have received little direct attention. This study directly confronts this gap by investigating the digital peripheral tie practices of urban Chinese older adults, with the goal of understanding whether and how the use of social media platforms opens up new paths to social support in later life.

## 2. Literature Review

### 2.1. The Fading and Digital Re-Emergence of Peripheral Ties in Older Adults’ Social Networks

As introduced earlier, the contraction of peripheral ties in later life is a phenomenon observed well before the digital era, explained by classical aging theories, and persisting as a consistent finding across decades of research. Over time, this understanding has effectively become a tacit assumption in the field. As a natural consequence, aging research has predominantly examined close relationships and the support they provide (Rook, 2015), with peripheral ties receiving little scholarly attention, leading to a remarkably limited understanding of how older adults’ peripheral ties may be evolving in the digital context. Nevertheless, two converging lines of evidence suggest that a digital re-emergence of these ties may be underway.

The first comes from research on the mechanisms through which digital platforms reshape social connectivity for their users. Research has highlighted that these platforms allow people to stay in touch with contacts regardless of physical distance, meaning that geographic isolation no longer has to lead to social disconnection the way it once did, particularly for older adults (Antonucci et al., 2017). Moreover, in digital networks, peripheral ties do not have to disappear entirely. They can sit alongside close relationships in a kind of dormant state, still visible and reachable even if not actively maintained (Bayer et al., 2020; Haythornthwaite, 2002). Online environments also make it possible for people to form entirely new weak connections, the sort that would less readily develop through face-to-face interaction alone (Bayer et al., 2020). These mechanisms jointly suggest that peripheral ties that have gone unattended in older adults’ social lives need not disappear from their social world entirely, and that their peripheral networks may remain open to newcomers through digital interaction.

The second line of evidence comes from empirical studies that have begun to document positive associations between older adults’ digital engagement and their broader social networks. A systematic review of the association between digital technology use and social capital among older adults found consistent evidence that digital technology use is positively related to both bridging and bonding dimensions of social capital in this population (Grotto & Buja, 2025). At a more granular level, research has demonstrated that social grooming behaviors on social media, such as liking and commenting on posts, are linked to higher bridging social capital among older adults, which in turn sequentially predicts greater perceived social support and life satisfaction (P. L. Liu & Yeo, 2024). These findings are significant because bridging social capital is precisely the type of resource that flows through peripheral ties (Granovetter, 1973).

Taken together, these two converging lines of evidence, one mechanistic and one empirical, suggest that older adults’ peripheral ties may indeed be sustained and even reinvigorated through their engagement with digital platforms. However, a critical gap remains. Current aging research has primarily documented the mere persistence of these ties, without unpacking their specific makeup. A clear, bottom-up typology reflecting the unique life-course characteristics of older adults is still lacking. To address this empirical gap, this study asks:

**RQ1.**  *In the digital environment, what peripheral ties do older adults actually maintain?*

### 2.2. Lightweight Digital Interactions and the Maintenance of Peripheral Ties

Maintaining large-scale peripheral networks offline demands continuous energy and cognitive capacity (Dunbar, 2016; Granovetter, 1973). However, the technological affordances of social media have substantially lowered this relational threshold. Through features like asynchronicity and the persistence of status updates, digital platforms alleviate the spatiotemporal pressure of real-time exchange (Treem & Leonardi, 2013). This restructuring provides the structural potential for lightweight digital interactions—enabling users to sustain connections with minimal effort.

In concrete digital practice, these lightweight interactions generally take two parallel forms. The first relies on ambient awareness, often conventionally labeled as “passive” observation (Levordashka & Utz, 2016). However, lurking—defined as reading without posting or commenting—has long been recognized as a normative and intentional pattern of online participation rather than an absence of engagement (Nonnecke & Preece, 2000). This approach allows users to produce psychological connection through substitute interaction—a mechanism documented across diverse online communities, including social support forums, wherein observing others’ exchanges generates psychological connection and vicarious social experience without requiring direct participation (Dai & Shi, 2022), entirely bypassing the burden of bidirectional reciprocation. The second form is proactive social grooming, comprising one-click likes or short standardized emojis (Burke et al., 2010). Through these preset symbols, users actively send relational signals to bidirectionally acknowledge a tie, replacing the mental expenditure of composing lengthy texts (Stsiampkouskaya et al., 2023).

When examining how older adults navigate these lightweight pathways, the existing literature highlights a dual reality. On one hand, older adults are frequently observed adopting a cautious approach, relying heavily on ambient awareness and passive observation (Lüders & Brandtzæg, 2017). On the other hand, research explicitly demonstrates that they also purposefully engage in proactive interactions (e.g., liking) to build bridging social capital (P. L. Liu & Yeo, 2024).

While passive observation and proactive interaction clearly coexist in older adults’ digital practices, existing studies rarely weigh their relative prevalence in the specific context of outer-circle networks. It remains entirely unclear which approach predominates when older adults manage their non-intimate connections. This ambiguity leads directly to the second research question:

**RQ2.**  *How do older adults deploy specific lightweight platform practices to maintain their digital peripheral ties, and do these practices vary depending on the specific type of tie?*

### 2.3. Functional Benefits and Emergent Forms of Support from Digital Peripheral Ties

A foundational proposition in social network research holds that strong ties primarily provide emotional support (bonding capital), whereas weak ties deliver novel information and diverse perspectives (bridging capital) (Granovetter, 1973; Putnam, 2000). Digital platforms provide particularly fertile ground for this informational function (Ellison et al., 2007). Specifically, platform-mediated architectures aggregate individuals from diverse backgrounds, substantially amplifying access to heterogeneous information flows that close personal networks cannot supply (Wellman et al., 2001, 2003).

Yet, the canonical boundary between informational weak ties and emotional strong ties is becoming permeable online. As the functional division between tie types grows increasingly complex, the theoretical possibility arises that peripheral ties might cross over into the affective domain under certain conditions, even as evidence on the extent of this crossover remains mixed (Krämer et al., 2021; Rozzell et al., 2014). Indeed, relationally non-close individuals in digital environments can provide levels of social support comparable to those of close ties (Rozzell et al., 2014). This is particularly evident when strong ties are unavailable or when managing sensitive issues, where weak ties are frequently sought out as valued sources of support (Moreton et al., 2023). Together, these findings suggest that digital platforms enable peripheral ties to function as meaningful sources of support beyond mere information brokerage.

Despite this emerging evidence, the aging literature presents a pronounced gap. While systematic reviews confirm that social media engagement correlates with positive psychosocial outcomes in older adults, the specific relational mechanisms producing these outcomes remain critically underexplored (Lei et al., 2024). Prior research on late-life social support has largely concentrated on familial support and formal institutional care (Rook, 2015). Beyond these two traditional poles, the functional role of digitally maintained peripheral ties has received scant empirical attention. It remains underspecified whether the benefits of these ties are strictly restricted to informational utility or extend into emotional or other broader psychological dimensions. This gap constitutes the third core question of the present study:

**RQ3.**  *What functional benefits do digitally maintained peripheral ties provide to older adults?*

## 3. Method

### 3.1. Research Design

**Figure 1:**
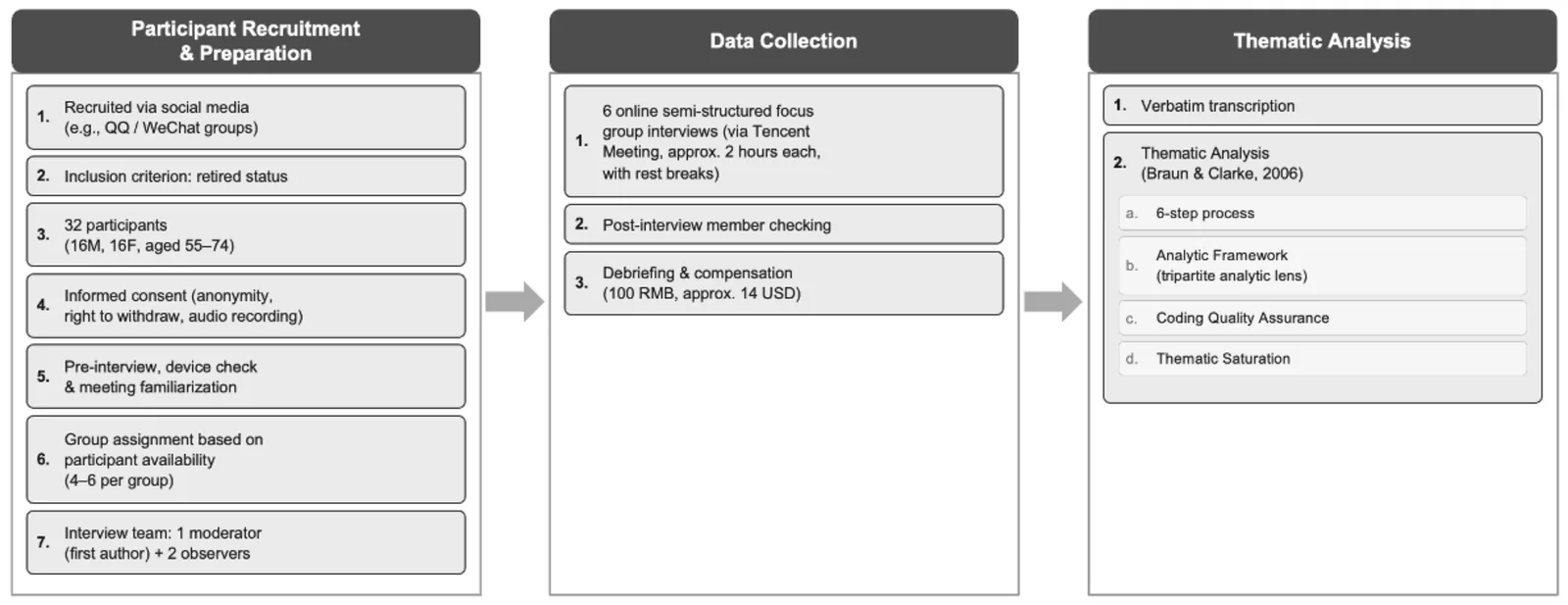
Overview of the Methodological Workflow (Braun & Clarke, 2006).

This study adopts an exploratory qualitative research approach to investigate in depth the subjective experiences, motivations, and behavioral processes of older adults in their digital social interactions. We selected WeChat as the research context because it is the most dominant and ubiquitous social media platform in China. Its core functionality supports a diverse range of interpersonal connections (Shen & Gong, 2019), and it is deeply embedded in the daily lives of the older population, thus serving as the de facto environment for observing their digital social interactions. Therefore, this study focuses on older adult users of the WeChat platform. This research design is conducive to capturing the rich details and complexities behind the aforementioned processes, thereby providing in-depth descriptive insights for the field. The methodological workflow of this study is summarized in Figure 1 (detailed in Section 3.2 and Section 3.3 below).

### 3.2. Participant Recruitment and Data Collection

This study primarily targets retired individuals as representatives of older adult social media users. The retired status, rather than a specific legal age (e.g., 60 years or older), was chosen as the core inclusion criterion based on the following rationale: retirement is typically associated with withdrawal from the formal workforce and a shift in life focus, posing a practical challenge of social network reconstruction for this group. Therefore, compared to the legally defined age, this criterion better ensures that participants align with the study’s core focus (i.e., the changing trends of social networks). The retirement defined in this study includes both formally retired individuals and those who have voluntarily withdrawn from business operations, all of whom have entered or are approaching old age and whose life focus has shifted to their personal lives.

This study collected data through online semi-structured focus group interviews (Stewart & Shamdasani, 2017; Tuttas, 2015). Focus group interviews were employed, rather than individual interviews, because the interactive environment of focus groups effectively encourages participants to share collective norms and shared meanings formed in their use of social media. Discussions and exchanges among participants are more conducive to revealing social conventions that individuals take for granted and that are not easily detected in individual interviews.

Six focus group interviews were conducted with a total of 32 older adult participants (16 male, 16 female), ranging in age from 55 to 74 years, with 5 to 13 years of WeChat experience (see Table 1 for an overview of participant groups; see Table S1 in the Supplementary Materials for detailed participant background information). All interviews were conducted via Tencent Meeting software (known internationally as VooV Meeting, version V3.39.0 or later), moderated by the first author, and observed by two research assistants trained in social sciences.

This study primarily recruits participants through recruitment advertisements on social media platforms such as QQ groups and WeChat groups. In the actual recruitment process, because the participants belong to a specific group, they primarily obtained the advertisements through information forwarded by their younger relatives or someone else before participating in the study. Participants had no personal connections with the researchers or research assistants. They were totally strangers to each other, and the same group of participants lived in different cities. No participants withdrew after recruitment, and no individuals who met inclusion criteria declined participation. 

The focus group discussions revolved around the elderly participants’ WeChat social experiences, their perceptions of online social connections, interaction styles, and perceived value (see Table 2). These questions were designed to elicit rich accounts of participants’ peripheral tie typologies, maintenance strategies and platform practices, and perceived functional benefits. The focus group discussions lasted an average of approximately two hours, with two to three breaks included.

To ensure the data quality and trustworthiness of the online interviews, this study implemented rigorous procedural controls: (1) Before the formal interviews, each participant underwent an initial participant check-in and an online dialogue practice session; (2) Rest breaks were set between interview stages, allowing the moderation team to organize key records and dynamically adjust subsequent questions; (3) After the interviews, individual follow-up visits were conducted with each participant for data verification (member checking) (Lincoln & Guba, 1985) and to obtain supplementary information. Upon completion of the follow-up, each participant received a full debriefing and was compensated with 100 RMB (approximately 14 USD). All interviews were fully audio-recorded with participant consent and subsequently transcribed verbatim for analysis.

### 3.3. Data Analysis

#### 3.3.1. Analytic Framework: Relational Awareness, Maintenance, and Functional Outcomes

The thematic analysis was guided by the core inquiries of this study, adopting a tripartite analytic lens. This framework directed coding attention toward three interconnected dimensions: (a) relational awareness and typology, focusing on how older adults perceive, make sense of, and categorize their varied peripheral connections; (b) relational maintenance, encompassing the operational strategies and lightweight platform practices used to manage these ties, as well as the perceived efforts or “costs” involved (e.g., time, energy, cognitive load, and social risk); and (c) functional outcomes, capturing the informational, emotional, and broader psychological benefits derived from these digital interactions. Consistent with (Braun & Clarke, 2006) approach, this framework served as a guiding lens for theme generation without imposing a priori categories on the data: codes and themes emerged inductively from participants’ own narratives, and this tripartite framework supported the organization and interpretation of those themes once they had been generated.

#### 3.3.2. Analysis Process

This study employed Thematic Analysis (TA) to analyze the data (Braun & Clarke, 2006), aiming to identify and organize core themes from the interview data. The analysis followed a hybrid path: it was both inductive (themes emerged bottom-up from the data) and theory-guided (the analysis was guided by the core concepts in our research questions, specifically the peripheral tie typology, maintenance costs and platform practices, and functional benefits). We strictly followed the six-step process proposed by Braun and Clarke (2006): (1) familiarizing with data; (2) generating initial codes; (3) searching for themes; (4) reviewing themes through two rounds to ensure internal consistency and clear boundaries; (5) defining and naming themes; and (6) producing the report. Themes, sub-themes, and representative coding examples are summarized in Table 3.

#### 3.3.3. Coding Quality Assurance

The first author of this study (responsible for primary coding) is a young person, whose generational background differs from that of the participants. While this helps maintain an outsider’s objective perspective, the researcher also recognized a potential positive assumption of expecting to find benefits. To counterbalance this potential bias and ensure the rigor and trustworthiness of the analysis (Lincoln & Guba, 1985), our coding quality assurance involved two levels. First, during the coding phase, two research assistants acted as coding supervisors (peer debriefing), holding regular meetings to review theme generation progress. Second, after the initial analysis, the second author, who shares a similar background with the participants, conducted a quality check from a user’s perspective (reflexivity), reviewing the generated themes for deviations from practical experience.

#### 3.3.4. Thematic Saturation

This study used thematic saturation as the criterion for determining the sufficiency of data collection (Guest et al., 2006). Saturation was considered achieved when new data no longer produced new themes or mechanisms, and the relationships between existing themes became stable. By the fifth focus group, no new themes emerged; the sixth group confirmed existing patterns.

## 4. Findings

**Figure 2:**
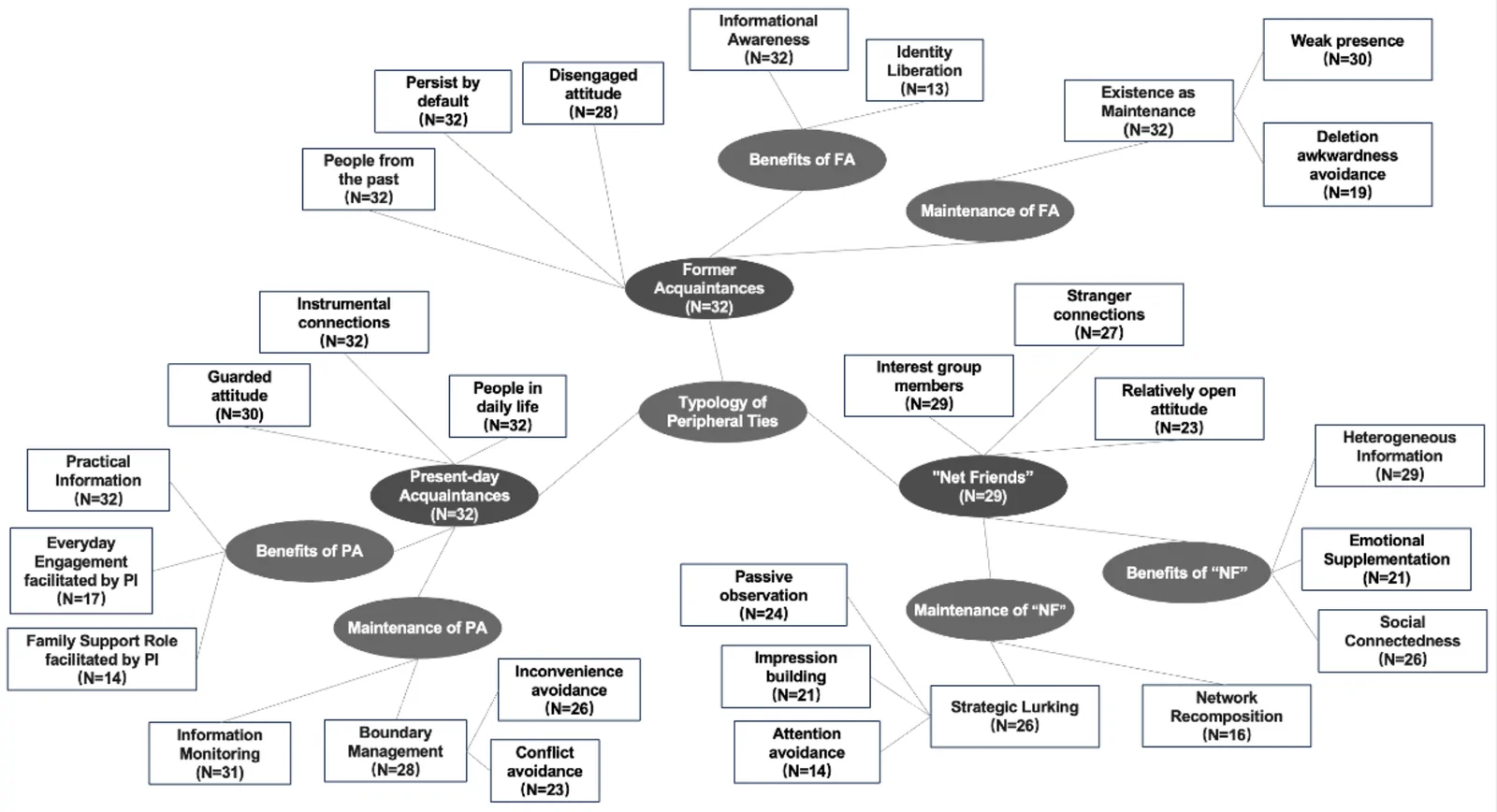
Detailed Thematic Map of Older Adults’ Digital Peripheral Tie Practices. *Note.* FA = Former Acquaintances; PA = Present-day Acquaintances; NF = “Net Friends”; PI = Practical Information. N = number of participants who explicitly mentioned or confirmed the presence of the concept during focus group sessions (out of 32). These figures are descriptive summaries provided for distributional transparency.

To provide an overall picture, a detailed thematic map illustrating the full thematic structure, including sub-themes and lower-level codes, is presented in Figure 2, where each of the three tie types can be traced as a complete profile across all three dimensions. The map is organized by tie type to show each type’s complete profile across all three dimensions. The sections that follow, however, are organized to address the three research questions progressively. We first present the three-category typology of peripheral ties (RQ1), then examine the differentiated maintenance strategies for each type (RQ2), and finally document the multidimensional functional benefits these ties yield (RQ3).

### 4.1. Theme One: A Three-Category Typology of Peripheral Ties on WeChat (RQ1)

Older WeChat users perceive and categorize their non-intimate contacts into three functionally and attitudinally distinct types: Former Acquaintances, Present-day Acquaintances, and “Net Friends.” Structurally, these ties exist in two forms: as individual contacts in the friend list, or as fellow members within WeChat groups.

#### 4.1.1. Former Acquaintances: Passive Retention Amid Attitudinal Disengagement

Original relationships that have faded due to shifts in life focus or the passage of time—such as former colleagues, teachers, or old neighbors—are referred to by older adults as “people from the past” (以前认识的人). Structurally, these Former Acquaintances exist both as individual contacts and within legacy WeChat groups (e.g., alumni or former-workplace groups). Because they no longer hold active informational or emotional value, participants adopt a passive attitude: these ties persist by default, without active investment or deliberate severance.

*Participant 14 described this state matter-of-factly: “On my WeChat I have not only former colleagues, but also former teachers and classmates, and I think a few neighbors from before I moved. That’s pretty normal. Having someone on WeChat doesn’t mean we have to be in touch. Going a long time without contact is normal too. For some of them I never updated the contact note, and I don’t even remember who they are anymore. Keeping acquaintances on WeChat is just more convenient than a phonebook.”*

*Reflecting on former colleagues, Participant 12 stated, “My life focus is now entirely on myself; I don’t care what’s happening at the old workplace, unless they happen to distribute extra retirement benefits.” Participant 11 added, “Some classmates were fine back in school, but people change, and it feels awkward to chat on WeChat once the relationship has faded.”*

This disengaged attitude, however, coexists with a quiet recognition that the mere presence of these contacts carries a faint psychological weight.

*Participant 7 described a similar feeling about old classmates: “Sometimes I flip through the contact list and see those old classmates’ names; it’s like flipping through a photo album. I don’t message them. I just know they’re there, and that feels different from not having them on my list at all.”*

#### 4.1.2. Present-Day Acquaintances: Contacts and Groups from Current Daily Life

Contacts acquired through current daily routines—such as community workers, activity organizers, or local retailers—are termed “people I know now” (现在的熟人). Present-day Acquaintances appear as individual contacts or within utility-driven local groups (e.g., neighborhood discount groups). Unlike groups composed of Former Acquaintances, these utility groups typically range from dozens to hundreds of members, the majority of whom are strangers. Participants approach these ties strictly for practical utility, prioritizing instrumental value and deliberate boundary management over relational depth.

*Participant 22 noted that her most frequently opened WeChat groups are local retailer discount groups joined via offline QR codes. She explained that “these groups contain both store sellers and other ordinary consumers like myself,” and although “none of us know each other,” it does not stop members from “forwarding all sorts of local store promotions they happen to come across, helping each other snag deals.” Similarly driven by utility, Participant 23 mentioned joining walking groups “just to receive messages… if I talk too much, it might lead to unpleasantness, which is just causing trouble for myself.”*

This conservative approach is heavily reflected in social risk avoidance.

*Participant 21, referring to a fishing enthusiast group, said he “only discusses fishing or other ‘innocuous’ things,” believing that in a group of “thirty or forty people who barely know each other,” saying the wrong thing could cause real trouble. Participant 14 mentioned segregating all “useful people,” such as property managers and couriers, adding notes to their profiles so “I can quickly find them if I need anything,” while ensuring “they can’t see my Moments.”*

#### 4.1.3. “Net Friends”: Members of Interest-Based WeChat Groups

The third category, “Net Friends” (网友/群友), refers to co-members of interest-based WeChat groups. Unlike the first two categories, Net Friends appear almost exclusively as fellow group members rather than individual contacts. Participants build these networks primarily through two pathways: joining reader groups linked to WeChat Official Accounts, or scanning QR codes found on other platforms. Compared to the other two types of ties, participants’ attitudes toward Net Friends are relatively open and engaged.

*Participant 7, who spends her retirement traveling and tending plants, follows many gardening and travel Official Accounts. She mentioned her WeChat groups are mainly attached to these accounts, plus groups “recommended by fellow members from the existing groups.” All of them, she said, are populated by people who share her interests.*

*Participant 6 is a member of several stock-trading groups. He joined these by scanning QR codes posted in the comment sections of stock investment forums and finance-themed video accounts on other platforms. He noted that some original groups had “too many scammers,” prompting him to actively withdraw and “keep only those where most members appeared to be ordinary retail investors.” In those remaining groups, he mostly observes other members exchanging insights.*

In summary, older adults maintain three structurally and attitudinally distinct categories of peripheral ties on WeChat, a typology previously unidentified in aging research.

### 4.2. Theme Two: Differentiated Maintenance Strategies by Tie Type (RQ2)

#### 4.2.1. Former Acquaintances: “Existence as Maintenance”

For Former Acquaintances, participants employ a passive retention strategy: the continued existence of these contacts in the WeChat list is itself the maintenance. Participants do not initiate interaction and actively avoid engagement, yet they also do not delete these contacts. The rationale is twofold. First, the social risk of deletion (the potential embarrassment if the other contact notices the removal) outweighs the negligible effort of retention. Second, the contact list’s persistence affordance allows these ties to exist indefinitely without any relational expenditure. Occasional passive exposure occurs when participants receive broadcast messages (such as holiday greetings), but these are noted and dismissed rather than reciprocated.

*Participant 10 said, “I actually wanted to delete them (former colleagues) long ago, but I was worried it might be awkward, like if someone sends a holiday greeting and realizes I’ve removed them.” Participant 14 suggested an alternative to deletion: “No need to delete them. You can just swipe left on the chat and select ‘Do Not Show,’ so the chat stops appearing in your conversation list, and pretend they’re not there. For the group chats containing these old colleagues or classmates, you can simply ‘fold the chat’ so it stops appearing at the top of your chat list.”*

This passive existence, however, is a subtle one, governed by its own implicit rules.

*For example, Participant 9 said he did not mind retaining these past ties as long as “neither side’s presence felt too strong.” He recalled, “One time, a classmate I hadn’t been in touch with for years asked to borrow money—refusing would hurt our relationship, but if I lent it, I doubted I’d ever see that money again…I thought to myself, it would be better if he hadn’t realized he was on my WeChat, or if I wasn’t on his.” Participant 12 raised a related concern about former colleagues remaining on her contact list: “I try to avoid any contact with them on WeChat, in case someone mistakenly thinks we’re still close and then suddenly sends me a wedding invitation for their child…that’s not really appropriate…some colleagues have a better mutual understanding—we haven’t deleted each other, but we don’t bother each other either.”*

#### 4.2.2. Present-Day Acquaintances: Information Harvesting with Boundary Management

For Present-day Acquaintances, the dominant strategy is systematic information monitoring combined with deliberate social boundary management. Participants actively read group messages (discount announcements, service notices, activity information) but consciously suppress any behavior that might signal relational investment or create expectations of reciprocity. As illustrated by Participants 22, 21, and 14 in the typology section above, this strategy involves reading for utility, speaking only on safe topics, restricting Moments visibility, and adding notes for rapid retrieval. 

Notably, participants placed strong emphasis on boundary-setting because “crossing” these boundaries could bring inconvenience or even risk.

*Regarding inconvenience, Participant 13 described a tutor he had added on WeChat after enrolling his grandson in an after-school class. As school breaks approached, the tutor would frequently like his Moments posts and send him advertisements for the class. On one occasion, he told the tutor directly that his grandson would not be visiting that break; when his grandson ended up visiting after all, he felt too embarrassed to post about it on Moments, worried the tutor would see it and think he had lied. Participant 14 said he fully understood this awkwardness, explaining that he avoided letting such “instrumental contacts” see his Moments in order to prevent personal or family-related information from becoming someone else’s “business opportunity.”*

*Regarding risk, Participant 23 explained her reluctance to speak up in groups by recounting an incident in a group she belonged to, where a disagreement escalated into a physical altercation and injury during an offline gathering. She reasoned that “some people are quite stubborn…if they can’t win an argument in the group, they’ll want to ‘settle’ it offline in person, and getting entangled with someone like that is a lot of trouble.” Participant 21 agreed strongly, adding, “since there’s a possibility of a negative impact on real life, why would [we] want to risk that?”*

Taken together, the maintenance effort is minimal—essentially the cognitive bandwidth required to filter group messages and demarcate boundaries—while the instrumental yield is tangible and immediate.

#### 4.2.3. “Net Friends”: Strategic Passive Lurking and Network Recomposition

The maintenance of “Net Friends” centers on strategic lurking (covert observation), coupled with network recomposition. This dual approach offers low relational friction, minimal social risk, and sustained engagement.

*Participant 8 mentioned that her daughter often calls her a “super lurker”, “hiding in all sorts of WeChat groups.” She characterized this lurking not as intentional disengagement but as organic familiarization: “Just like staying in an environment for a long time, you observe the environment, and eventually you get familiar with these people… I’m usually spot on with my guesses.”*

Lurking also functions as a deliberate risk-management strategy, as participants avoid leaving an impression that would constrain their later freedom to disengage.

*Participant 5 reflected, “If I’m too active and leave an impression, it would be a bit embarrassing if I stopped talking later or wanted to quit for some reason.”*

Participants further reported that their observation extends to viewing individual group members’ profiles to build cognitive impressions, without initiating direct contact.

*Participant 6 articulated this explicitly: “Sometimes I’ll open someone’s profile in the group to see what region they’re from, what they’ve posted in their Moments. You form an impression. But I wouldn’t send a friend request. That feels like crossing a line.” Several other participants endorsed this practice as their own routine.*

In contrast, active approaches (speaking or interacting) frequently generate energy burdens.

*Participant 18 confessed, “I could speak, but I always feel like there’s too much to say; it’s exhausting, so I just let it go.” Participant 17 commented, “At this age, there’s no need to argue with people; it’s too much hassle. Although I didn’t say anything, I’ve already refuted it in my mind.”*

Because active rescue efforts for silent groups are cognitively consuming and often unsustainable, participants favor network recomposition over relational repair.

*Participant 11 described his failed attempt: “I began throwing out topics to ‘save’ the group, but it was quite consuming, and I’m not sure if I would do it again.” Participant 15 asked rhetorically, “What’s the point of staying in a group where no one talks?” This leads to a pragmatic resolution, as Participant 16 concluded: one should “take it easy” because “there’s always another way forward” (*东边不亮西边亮*). Defunct groups are simply replaced with new ones, sustaining the structural presence of “Net Friends” without burdensome attachment to any specific group.*

In summary, older adults apply three qualitatively distinct maintenance strategies across the three tie types. Former Acquaintances require zero active maintenance; Present-day Acquaintances are managed through information monitoring and boundary management; and “Net Friends” are sustained through strategic lurking and network recomposition. All three strategies are governed by an overarching principle of energy conservation and relational pragmatism.

### 4.3. Theme Three: Multidimensional Functional Benefits of Digital Peripheral Ties (RQ3)

#### 4.3.1. Former Acquaintances: Informational Awareness and Retrospective Identity Liberation

Although participants hold disengaged attitudes toward Former Acquaintances, these ties are not devoid of functional yield.

First, their passive presence provides a low-stakes informational awareness. Despite general disinterest in news from former workplaces or schools, participants prefer to remain loosely informed. This is driven less by the information’s actual utility and more by social image maintenance—specifically, the wish not to appear out of touch with their former group.

*Participant 26 explained this underlying motivation: “I don’t usually care about news from my former office, but if something happens, I’d rather see it first and then decide it’s not important, than find out I’m the only one who didn’t know. If others bring it up and I have no idea, they might tease me for being “completely disconnected from everything” (*两耳不闻窗外事*). I don’t want to be seen that way.”*

Second, and more subtly, retaining these contacts affords a distinctive retrospective identity liberation: the sense of relational autonomy that comes from holding once-binding ties at arm’s length. In the pre-retirement era, interactions with peer colleagues were structured by hierarchical obligation and the careful management of face. Retirement does not erase these relationships, but WeChat’s persistence affordance allows them to exist drained of obligation, offering a quiet sense of freedom.

*Participant 25, a former leader before retirement, described this dynamic: “Back when we were all working together, every interaction with colleagues required careful thought, what to say, what not to say. Now we’re all retired. Their names are still in my WeChat, but I don’t need to greet them or worry about how I come across. It’s actually a nice feeling. These old relationships are still there, but they don’t hold me anymore.”*

#### 4.3.2. Present-Day Acquaintances: Practical Information, Family Support Role, and Renewed Social Participation

Present-day Acquaintances yield the most immediately tangible functional benefits, operating along three interconnected dimensions.

First, these ties provide practical informational value. Participants receive a steady stream of actionable information about local commercial and community environments, significantly enhancing daily life convenience.

*Participant 22 captured this concisely: “I can see discount information about pharmacies, supermarkets, and department stores (from both store sellers and ordinary consumer members in these groups); it’s very convenient.”*

Moreover, this convenience encourages older adults to get out and engage in everyday social life.

*Participant 24 mentioned that although she has help with shopping at home, she still joins local shopping groups, because she can “use discount information as an excuse to go out with my husband. It’s exercise, and it gives me a sense of being part of social life.” Participant 25 noted, “There’s a free photography exhibition in our community recently. I saw it in a staff member’s Moments post, so I went to have a look. It feels good to still be out and about, interacting with people.”*

Second, this informational yield extends beyond self-care to sustain a family logistics support role. Older adults frequently relay local deals or service details to their busy adult children, reinforcing their sense of continued social usefulness.

*Participant 20 described sharing group information with her daughter and son-in-law: “They are always at the office and have no idea about these neighborhood deals. When I spot something useful in the group and send it to them, I feel like I’m still doing something useful for the family, that I haven’t been set aside.” Participant 16 added: “When my daughter asks me, ‘Mom, where did you hear about that?’… and I say ‘from a group,’ she’s surprised. It makes me feel like I’m not falling behind, and that I can still help her out.”*

Notably, this sense of usefulness did not appear to depend on the child’s response—the act of relaying itself carried meaning.

*Participant 15 recalled sending his son items he thought were useful, only to get no reply, or just an “oh”: “I don’t even know if he’s read it…but it doesn’t really matter. Sending it means I’ve done my part, shown that I care.” Participant 17 described his child characterizing a shared event as merely the neighborhood committee’s “political achievement” project, cautioning him not to trust such things, yet added, “His view might be different from mine, but I’ll still send it—it’s a form of communication after all…who knows, maybe the next one will actually be useful.”*

Taken together, these benefits generate a third, overarching dimension: a renewed sense of ongoing social participation, which counters the potential disengagement that retirement can otherwise impose.

#### 4.3.3. “Net Friends”: Heterogeneous Information, Emotional Supplementation, and Broad Social Connectedness

“Net Friends” yield three distinct benefits, the first of which is heterogeneous information. Because these groups gather members who share a specific interest yet otherwise have little real-world overlap and markedly different backgrounds, the perspectives circulating within them are diverse and non-redundant, exposing older adults to viewpoints that their close, largely homophilous networks rarely supply.

*Participant 9, recounting a foreign-language study group, said the discussion angles on the international affair news were “all over the map, and quite reasonable,” offering “many perspectives I hadn’t thought of,” which he found “very rewarding.” For Participant 10, such groups are “really valuable… they encourage us to listen and observe more and keep us from getting stuck in our ways,” guarding against being dismissed by younger people as “too old-fashioned.”*

The second benefit is emotional supplementation. In contrast to the cognitive diversity that drives the informational value, this affective yield arises from the opposite mechanism—a strong overlap of interests and the resonance it produces. Through shared enthusiasm and the steady, low-pressure co-presence of fellow members, “Net Friends” groups furnish companionship, belonging, and a buffer against loneliness, even in the absence of direct interaction.

*Participant 7, a member of a travel-themed reader group, contrasted the group with her family, who “won’t travel with me, and they criticize photos I post on Moments as ‘dated’.” Among the friendly and knowledgeable group members, by contrast, “the emotional value is maximized, making me feel my sharing is meaningful”; sometimes, she said, the group “feels more like ‘one big happy family’ (*相亲相爱一家人*).”*

For others, this companionship is actively sought.

*Participant 8, who lives alone, “saves up” messages from a few groups to read at night before bed, so that she can take in “more messages at once” and “feel like ‘everyone is here with me,’” which makes her “not so lonely.” Participant 6 likewise keeps a few “curated” groups as a form of “spiritual sustenance” when his mood is low, watching the exchanges of active members “like watching a comedy show.”*

The third benefit is broad social connectedness. Distinct from heterogeneous information, which concerns the diversity of content older adults take in, broad social connectedness concerns their felt relationship to the wider social world—a sense of remaining plugged into authentic, real society rather than receiving isolated facts. While family networks are constrained by homophily and time, WeChat groups offer a direct, authentic link to broader society. Participants contrasted the richness of WeChat groups with the sterile environment of news apps: while apps deliver isolated facts, WeChat groups deliver public sentiment grounded in identifiable, real people.

*Participant 28 expressed this pointedly: “Some of the comments under web news are clearly “paid trolls” stirring things up, or bots writing nonsense. After reading too many of those, you lose your sense of judgment. In the groups, the people forwarding and discussing are real.” Participant 30 added, “On the news app, I can read what happened. But in the group, I can also see what everyone thinks about it… That feels like real life.”*

In addition, this broad social awareness translates into offline “conversational capital”: it equips older adults with rich material that lubricates everyday exchanges within their strong-tie networks, whether chatting with a spouse, old friends, or younger family members.

*Participant 31 provided a vivid example of how this information flow prevents her from feeling marginalized in family conversations: “They don’t have that much time to sit and chat with me about everything that’s going on… But in these groups, information just flows in. And then when I sit down with my grandchild, I actually have interesting things to say. I don’t sound like an out-of-touch old person.”*

In summary, RQ3 is answered. The peripheral ties maintained by older adults on WeChat yield a multidimensional benefit structure. Former Acquaintances provide low-stakes informational awareness and retrospective identity liberation. Present-day Acquaintances deliver practical information that supports self-care, social participation, and a family logistics support role. “Net Friends” ties offer heterogeneous information, emotional supplementation through vicarious participation, and broad social connectedness that provides conversational capital for offline relationships. More importantly, across all three tie types, the ultimate psychological payoff converges on the emotional dimension: even the most instrumentally framed informational benefits ultimately resolve into experiences of self-worth, competence, belonging, and relational meaning.

## 5. Discussion

This study advances the empirical and theoretical understanding of how older adults manage and benefit from peripheral ties in the digital era. By identifying a three-category typology, documenting their differentiated maintenance strategies, and mapping their distinct functional contributions, the findings provide the first systematic, empirically grounded account of older adults’ peripheral networks on a major social media platform.

### 5.1. The Typological Reconfiguration of Older Adults’ Peripheral Ties on WeChat

The three-category typology proposed in this study—Former Acquaintances, Present-day Acquaintances, and “Net Friends”—constructs a bottom-up classification of older adults’ digital peripheral relationships. This moves beyond the diffuse, largely undifferentiated view of online peripheral ties prevailing in prior aging literature (Bayer et al., 2020; Grotto & Buja, 2025; Jung & Sundar, 2016; P. L. Liu & Yeo, 2024), providing a structured map to examine relational dynamics in later life.

When viewed through the Social Convoy Model (Kahn & Antonucci, 1980), our findings confirm its classical prediction while illustrating its contemporary digital evolution. Consistent with the original model, the Former Acquaintances category shows that ties from pre-retirement settings (workplaces, schools) naturally fade in salience. On WeChat, these ties persist merely through structural retention and psychological disengagement, demonstrating how the traditional outer convoy loses density over time.

However, recent extensions of the convoy framework anticipate that life-stage transitions and digital platforms actively reshape older adults’ networks (Antonucci et al., 2017). Our findings empirically anchor this theoretical shift. The Present-day Acquaintances category demonstrates that as historical peripheral layers fade, new ones are simultaneously generated through post-retirement routines (e.g., community services, local discount groups). These contemporary ties step in to provide the routine integration and practical support previously supplied by the old outer convoy. In effect, the periphery is not simply shrinking; it appears to be replenished by current life circumstances.

The “Net Friends” category introduces a more novel form of convoy augmentation that expands the model’s original life-course assumptions. Although these contacts are geographically distant strangers, they provide persistent, low-threshold accompaniment through shared digital spaces and topical interests. This confirms recent calls to incorporate chosen, digitally mediated relationships into the convoy framework (Antonucci et al., 2017), shifting the anchor of convoy supplementation from physical co-presence to ambient digital visibility. However, our findings reveal that this digital layer goes beyond mere static supplementation; it exhibits a unique internal dynamism—a continuous iteration within the shifting network. Because these ties are untethered from physical geography or offline routines, older adults can effortlessly discard inactive groups and replace them with new ones (as evidenced by their “network recomposition” strategy). This transforms the outermost layer of the convoy into a highly fluid, self-renewing system—a dynamic fundamentally distinct from the steady accumulation or decline of offline ties.

Taken together, our findings suggest that within a digital ecology, the peripheral support circle of older adults is undergoing an active reconfiguration: old ties fade, present-day ties replenish the outer layers, and digitally native “Net Friends” provide a new form of ambient accompaniment. What older adults maintain is not the same outer convoy, but a recomposed one shaped equally by life-stage change and platform affordances.

### 5.2. ‘Passive’ Observation as Differentiated Agency: The Role of Relational Aggregation

Our study joins the ongoing dialogue regarding older adults’ social media practices—where both active and passive participation have been well documented (Lüders & Brandtzæg, 2017)—by exploring how they practically operate within the peripheral layer of their networks. Our findings demonstrate that for these peripheral ties, their engagement is predominantly “passive,” relying overwhelmingly on observation rather than interaction. However, this passivity is not uniform or disengaged; rather, it is highly differentiated. Older adults calibrate their observational modes precisely to the distinct relational logic of each tie type, transforming passive looking into a proactive, adaptive strategy of network maintenance.

Specifically, participants substitute observation for interaction across their peripheral network. Flipping through the contact list to register the suspended presence of various ties from the past (e.g., former colleagues, classmates, and previous neighbors), opening Present-day Acquaintance groups to scan for practical utility, and reading without speaking in “Net Friends” groups to absorb emotional texture: these are all deliberate forms of relational management enacted entirely through looking. This observational mode aligns closely with the concept of substitute interaction articulated by Dai and Shi (2022), wherein observing others’ exchanges functionally substitutes for direct participation, generating psychological connection and acquiring corresponding subjective experiences without the friction of expressive labor. Although Dai and Shi’s original study focused on health-related online support communities, the underlying mechanism of passive observation generating vicarious social experience appears to operate across different platform contexts, as evidenced by our participants’ accounts of reading group exchanges to feel present and connected without contributing directly.

This preference for observation over interaction also functions as an implicit safety strategy. Our findings suggest that, across all three tie types, when this strategy shifted—Former Acquaintances’ presence resurfacing unexpectedly, the “boundary” with a Present-day Acquaintance being breached, or overinvestment in a “Net Friends” group—such shifts could bring inconvenience or risk. This resonates with the sociological concept of symbolic boundaries (Lamont & Molnár, 2002), which describes the conceptual distinctions that groups draw between permissible and impermissible behavior within a given tie. The framework further holds that when symbolic boundaries are widely agreed upon, they can take on a constraining character. This implies that the boundary-maintaining strategies documented in this study, while serving as the primary means by which successful practitioners remain on the safe side of these implicit relational boundaries, may simultaneously carry an exclusionary dimension for those on the other side. Our data suggest that this exclusion may operate through at least two mechanisms. The first is relational withdrawal triggered by norm violation. When a long-dormant Former Acquaintance suddenly requested a loan (Participant 9) or a Present-day Acquaintance exploited Moments posts for commercial opportunity (Participant 13), the individual who unknowingly violated the implicit rule was never informed of its existence, but risked being cut off from the relationship or was directly excluded from information access (Participant 14). The second is self-silencing of dissenting perspectives. After witnessing a group disagreement escalate into a physical altercation during an offline gathering (Participant 23), participants chose to confine their interactions with Present-day Acquaintances to “safe topics” (Participants 21, 23) or refrain from debating with “Net Friends” (Participant 17), effectively isolating others from the diverse viewpoints they might otherwise have contributed. Together, these patterns suggest that the protective and exclusionary effects of symbolic boundaries are two sides of the same digital practice. The very strategies that shield older adults from relational risk simultaneously exclude norm-unaware contacts from information flows without explicit notice.

Notably, the viability of this observational strategy is predicated on a foundational, yet frequently overlooked, structural affordance: the capacity for persistent relational aggregation. Social media platforms spatialize and visualize relationships, congregating a vast array of peripheral ties into concrete, contained digital spaces—such as the contact list repository or group chat windows. In these spaces, both the contacts themselves and their ongoing interactions are structurally stored and continuously available for inspection (Bayer et al., 2020; Treem & Leonardi, 2013). Because the platform holds these ties in persistent visibility, observation alone suffices for relational maintenance. Without such aggregation, the same upkeep would demand far more active, effortful interaction.

Therefore, our findings enrich and concretize prior characterizations of “passive” media use among older adults, which often carry implicit connotations of low engagement. Recent work, such as Brewer et al. (2021) eye-tracking study, has begun to show that reading without commenting is intentional and relationally meaningful. Our study extends this insight by demonstrating that for the peripheral network, observational engagement is not merely a fallback for those lacking digital skills or a means to avoid intruding upon others; it can also be a proactive choice. It operates as a highly strategic maintenance mechanism that organically emerges from the online social environment. In essence, by leveraging the platform’s capacity to structurally aggregate ties, older adults turn passive observation into an active, differentiated mastery of their social networks.

### 5.3. The Emotional Functions of Weak Ties and Older Adults’ Emotional Priorities

Our findings show that the peripheral ties older adults maintain on WeChat deliver a range of benefits, spanning practical information, a family logistics support role, sustained social participation, emotional companionship through shared interests, emotional supplementation during moments of vulnerability, broad social connectedness, and offline conversational capital. It is worth noting that, although the benefits originate in functionally distinct domains, they converge, at their deepest level, on emotional well-being. We discuss these findings by engaging with two classic theoretical traditions.

First, our findings expand the classical weak-tie tradition (Granovetter, 1973) by highlighting the emotional functions of digital peripheral ties in the online context. The classical account posits that weak ties primarily deliver information and instrumental opportunities. This proposition found renewed empirical expression in early social media research, where bridging social capital—the access to diverse, non-redundant information through nonclose ties—was identified as a core benefit of online network participation (Ellison et al., 2007). At first glance, the Present-day Acquaintances category fits squarely within this account, providing local discount notices and service updates. However, our data reveals that the functional repertoire of digital weak ties extends far beyond mere information. The “Net Friends” category delivers emotional yield directly, buffering loneliness and providing companionship, which visibly extends weak-tie functions into the affective domain. This corroborates recent work showing that nonclose ties can provide meaningful social support online (Moreton et al., 2023; Rozzell et al., 2014). Furthermore, even the structurally dormant Former Acquaintances yield significant emotional relief through the sense of “retrospective identity liberation” and relational autonomy. In the digital ecology, weak ties have become rich sources of emotional and psychological value.

Second, these findings demonstrate the continued robustness of Socioemotional Selectivity Theory (SST) (Carstensen, 1992; Carstensen et al., 2003) within contemporary digital environments. SST argues that older adults prioritize emotional gratification, actively managing their networks to avoid negative affect and obligatory burdens. Our findings confirm that this emotional-priority principle remains the dominant driver of older adults’ online social practices; however, they reveal a novel pathway: older adults actively extract this emotional gratification from the periphery of their networks as well. Specifically, they achieve this through two distinct mechanisms. One is through the direct emotional benefits mentioned above (the low-threshold companionship of “Net Friends” and the relational autonomy of “Former Acquaintances”). The other, more subtle mechanism is the transformation of information into emotional currency. For instance, the practical information gained from Present-day Acquaintances is relayed to family members, allowing older adults to sustain their roles as useful family contributors. Similarly, the broad social awareness gained from “Net Friends” is used as offline conversational capital, underwriting their subjective sense of being engaged participants in social life rather than bystanders. As prior research suggests, sustaining these feelings of social usefulness and engagement is fundamental to older adults’ psychological health (Lei et al., 2024; Rook, 2015). Through these pathways, the acquired information is ultimately converted into self-evaluation, continued role identity, and relational meaning.

In short, our findings suggest that digital environments do not change the fundamental nature of older adults’ emotional priorities, but they substantially reduce the threshold and diversify the pathways for fulfilling them. By integrating persistent relational aggregation with their own strategic observation, older adults have effectively transformed the periphery of their social networks—once considered structurally “useless” or purely instrumental—into an important source of emotional support that sustains their psychological well-being. In other words, what emerges here is a lightweight yet meaningful support circle that complements the strong-tie core.

### 5.4. Structural Risks of the Lightweight Support Circle

The lightweight support circle, as fully discussed above, provides older adults with meaningful informational, emotional, and identity-related benefits. However, for this circle to function effectively and sustainably, it needs to remain open to diverse inputs. Our findings suggest that this openness may be constrained in three respects. 

First, interactions with Present-day Acquaintances are largely confined to “safe topics”, with contentious or controversial contents systematically avoided. The information that flows through these ties is thus restricted to a narrow bandwidth. Second, within “Net Friends” groups, participants usually adopt an observation-only posture, absorbing the perspectives contributed by others but rarely engaging through interaction or debate. While this allows them to receive diverse viewpoints, it may foreclose the possibility of testing, questioning, or building upon those viewpoints through active exchange, leaving their engagement with the information at a surface level. Third, because “Net Friends” groups are organized around specific interests, the width of information accessible mainly depends on participants’ own interests. For those whose interests are narrow, these groups may function as internally coherent but externally insular information environments, constituting the kind of information cocoons (Sunstein, 2006) that limit exposure to heterogeneous perspectives.

These three constraints suggest that the support circle, despite its demonstrated value, is susceptible to informational fragmentation, in which older adults’ actual access to information constitutes only a partial segment of the fuller picture available in the broader digital environment, potentially skewing their perceptions of the issues they encounter.

Moreover, the first two constraints carry an additional dimension. In both cases, older adults are not lacking in knowledge or judgment. They can be attentive and discerning observers and may have mentally formulated a counter-argument when controversial viewpoints emerge. Yet the implicit norms lead them to withhold rather than contribute their perspectives, effectively diminishing their role as active knowledge contributors within these digital spaces. This pattern resonates with concerns about epistemic injustice (Fricker, 2007), in which individuals’ capacity to contribute to collective knowledge is structurally undermined. 

It should be noted that these risks do not negate the substantial benefits of the lightweight support circle in this study, but they indicate that the circle’s low-cost, low-friction safety strategies carry structural trade-offs. Reducing these risks may require consideration at two levels. At the technological level, platform design could seek to broaden older adults’ information exposure without increasing the social cost that they are evidently keen to minimize. At the social level, however, these risks are rooted in older adults’ reasonable need for relational safety, and any intervention must ensure informational openness without compromising the relational comfort that makes the support circle sustainable in the first place.

### 5.5. Older Adults’ Digital Peripheral Tie Practices in Comparison with Those of Younger Populations

The preceding sections, through multiple theoretical lenses, discussed older adults’ digital peripheral-tie practices across three dimensions: typology, maintenance, and benefit. This section situates these practices within a broader comparative frame to clarify what they contribute to the study of digital social life. We compare our findings with research on younger populations, including college students and working adults, who constitute the demographic mainstay of most existing research on digital peripheral ties. This comparison helps further locate where our findings align with and depart from the mainstream literature, and, because later life is a stage that younger populations will themselves eventually reach, offers a view of how digital peripheral-tie practices may evolve across a longer life course.

Research on younger populations indicates that their digital peripheral ties are organized around specific social roles, maintained through the active signaling of presence, and drawn upon chiefly for instrumental ends. For example, undergraduate students (M = 20.4 years) in one study categorized their online contacts into strong ties, latent ties with casual acquaintances, and contacts with strangers (Ellison et al., 2011), while graduate students (M = 30 years, SD = 7.75) in another reported that their contact lists comprised as many as fourteen distinct audience categories, organized chiefly by role and origin, including family, coworkers, classmates from multiple educational stages, and Facebook-only friends (Vitak, 2012). Undergraduate students in a separate study (M = 20.1 years, SD = 1.64) actively used social media to stay in touch with high school acquaintances, so as to prevent the relationship from fading or being forgotten, thereby preserving a reserve of social capital that could later prove useful (Ellison et al., 2007). In workplace contexts, organizational newcomers (aged 21 to 24) deliberately used likes, comments, and other lightweight actions to build visibility with coworkers as a step in organizational socialization (Huang, 2019), while employees more broadly (aged 24 to 56, most between 27 and 40) reported that liking a colleague’s post safeguarded their standing within a career-relevant network (Zhu & Miao, 2021).

The older adults in this study depart from this pattern along the same three dimensions. Their peripheral ties are organized not by social role but by a compressed distinction between people from their past and people from their present. They maintain these ties mainly through passive observation rather than active engagement, with little emphasis on signaling their presence. And they draw on these ties for both informational and emotional benefit.

In terms of typological composition, compared to younger populations, the older adults in this study organized their digital peripheral ties in a markedly simpler, temporally based manner, though both groups’ typologies are heterogeneous. This simplification may reflect a reduction in the cognitive resources typically required to manage a differentiated network of ties (Dunbar, 2016) in later life. Organizing ties by time rather than role further suggests a heightened sensitivity to time. In classic theory (Carstensen, 1992; Carstensen et al., 2003), this heightened time sensitivity is understood as a key driver of relational selection among older adults. Our findings suggest that this sensitivity extends further, shaping how people cognitively organize their digital peripheral support circle as they enter later life.

In terms of maintenance strategies, compared to younger populations, the older adults in this study relied on a relatively passive, low-presence approach to maintaining peripheral ties, running counter to the active, presence-signaling approach typical of younger populations. This bears on a broader debate in the social media literature on active and passive use, which originated with research linking passive browsing to upward social comparison and envy (Verduyn et al., 2015), and extended into a claim that active use (e.g., posting, commenting, messaging) is more adaptive than passive use (e.g., browsing without responding), though evidence for this claim has been mixed (Valkenburg et al., 2022). Our findings contribute to this debate in two ways. First, they add a data point suggesting passive engagement can be a functional mode of digital practice among older adults. Second, older adults’ passive pattern, read alongside younger populations’ active pattern, points toward a possible resolution: what counts as adaptive engagement may depend less on being active or passive per se than on the specific relational target it concerns (e.g., peripheral ties or other ties) and, plausibly, the life stage at which it occurs (e.g., earlier or later in life).

In terms of functional benefits, the older adults in this study appear to derive broader benefits from their digital peripheral-tie practices than younger populations do, drawing both instrumental and emotional value, whereas younger populations’ benefits remain chiefly instrumental. This disparity points to the affordances that digital platforms make available for social engagement. The most recent perspective on these affordances holds that a psychological need (N) drives use of a platform feature (F) to the extent that the feature’s affordance (A) satisfies that need, an account known as the Needs-Affordances-Features (N-A-F) perspective (Karahanna et al., 2018). Considered through this perspective, the two populations do not differ substantially in their use of affordances and features, as both, shown above, are able to maintain their online peripheral ties in their own adapted ways. Their needs in this practice are also similar in nature, both instrumental. Where the two populations differ is in how strongly this need was felt. For younger populations, meeting this instrumental need tended to carry real stakes, such as safeguarding a career-relevant contact or avoiding appearing disengaged to coworkers (Zhu & Miao, 2021), where falling short risked a tangible cost. Among the older adults in this study, the instrumental need took the form of consciously seeking timely, useful information and content of personal interest, but satisfying it was rarely a “must.” We suggest that, given similarly instrumental needs and comparable use of affordances and features, the difference in benefit stems from this difference in intensity. This points to a possible pattern at the level of platform affordances: a stronger need may confine its fulfillment to the domain it targets, whereas a weaker need imposes less such constraint, leaving room for benefit to spill beyond it. This insight carries two implications. First, the N-A-F perspective could usefully incorporate need intensity to better account for how affordances support social engagement. Second, older adults’ pattern of drawing on platform affordances under weaker needs, relative to younger populations’ pattern under stronger ones, should not be read simply as an age-related decline, but rather as a kind of advantageous retreat, one that yields broader benefit by asking less of the platform.

Taken together, this comparison points to three sets of differences that track the life course. The organization of peripheral ties differs, from fine-grained, role-based categories among younger populations to a simplified, temporal distinction among the older adults in this study. The maintenance of peripheral ties differs similarly, from actively signaling presence among younger populations to a lower-presence, more muted mode of engagement among older adults. And the benefits drawn from peripheral ties differ as well, from largely instrumental value among younger populations to instrumental value increasingly mixed with emotional value among older adults. These differences highlight older adults’ heightened sensitivity to time in digital practice, the life-stage-contingent adaptiveness of social media engagement, and the constraining effect that the intensity of a specific need exerts on the benefit that a platform affordance yields.

### 5.6. Practical Implications

#### 5.6.1. Age-Friendly Platform Design

The integration and visualization of relationships within digital platforms serve as the essential infrastructure for supporting older adults’ peripheral social networks. Based on our findings, we suggest three targeted improvements for age-friendly platform design.

First, platforms should optimize the structural visibility of relationships to assist older adults in managing their extended networks. Enhancing contact list interfaces with custom classification or tagging tools would allow older adults to perform the relational auditing necessary to organize their digital social circles with clarity, thereby reducing the burden of managing a large number of peripheral ties.

Second, for information-dense group environments, platforms should prioritize features that support scannable engagement. Automated message digests, keyword highlights, or granular notification controls are essential to minimize cognitive load. These tools enable older adults to stay informed about relevant updates without the necessity of continuous, high-effort participation, aligning with their preference for observational engagement.

Third, platforms should proactively ensure a sufficient supply of peripheral connections to support social replenishment. Integrating discovery features based on geographic proximity or shared interests provides older adults with pathways to discover new communities. Such mechanisms are critical for helping older adults maintain an active social periphery. Moreover, if such discovery features extend beyond users’ existing interest domains, they could also help broaden the informational diversity available within the support circle, reducing the risk of the homogeneous information environments discussed above.

#### 5.6.2. Support for Digitally Excluded Older Adults

The platform design recommendations discussed above, however, chiefly benefit older adults who already have, or are in the process of forming, such a support circle. Beyond this group, some older adults may be excluded from it altogether by low digital literacy or cognitive decline. Like their peers, they too need ways to counter social disconnection and diminished well-being. For this group, we propose accommodations beyond platform design alone.

Older adults with low digital literacy—who may hold a social media account but remain unfamiliar with features such as message filtering or visibility settings—are less likely to have established such a circle in the first place. Community-based digital literacy programs or peer mentoring could help this group acquire both the observational strategies described in this study and the corresponding platform operations, such as folding a chat or restricting Moments visibility. Indeed, such programs and peer-mentoring relationships could themselves become an initial building block of such a circle, introducing new present-day acquaintances or “Net Friends” through whom participants might begin to practice the observational strategies described in this study. 

Older adults experiencing cognitive decline present a more complex case. Even if adult children can help perform certain actions on their behalf—such as adding former classmates as contacts or joining interest-based groups—thereby physically assembling a support circle, these older adults may still need to draw on certain cognitive capacities to obtain the benefits documented in this study. Meeting this group’s needs is therefore likely to require more sustained, caregiving-oriented support that falls outside the scope of platform- or family-based assistance, and represents an important direction for future interdisciplinary research.

### 5.7. Limitations and Future Research

This study has several limitations that suggest pathways for future inquiry. First, as our focus was on urban Chinese older adults, future research could examine these dynamics in rural or different cultural contexts, where norms of relational obligation and social support structures may vary. Relatedly, our sample was slightly skewed toward higher educational attainment and professional occupations (see Supplementary Table S1). Any future quantitative extension of this typology (e.g., via survey or social network analysis) should therefore pay careful attention to proportional sampling across educational and occupational strata. Second, while focus groups are effective for capturing collective norms, they provide less insight into highly individualized trajectories of social network management. Future work could employ longitudinal designs or in-depth individual interviews to track how these peripheral ties evolve over time. Third, our typology is empirically grounded in the high relational aggregation characteristic of WeChat. Future research could investigate how these relational configurations manifest across platforms with different affordance profiles. Such cross-platform comparisons would allow us to discern how varying degrees of relational integration shape the formation of peripheral ties, thereby enriching our understanding of how technical affordances—distinct from or similar to those of WeChat—support the construction of digital social circles.

## 6. Conclusions

This study provides a systematic account of how older urban Chinese adults construct, maintain, and derive benefit from a diversified portfolio of digital peripheral ties on WeChat. We identify three categories—Former Acquaintances, Present-day Acquaintances, and “Net Friends”—each managed through observational strategies. Far from being disengaged, older adults utilize these ties through strategically optimized observation, extracting multidimensional benefits: practical information, identity maintenance, and direct emotional support. Together, these peripheral ties form a lightweight, meaningful support circle. This circle may serve as a buffer against the social disconnection, diminished self-worth, and loneliness that often follow retirement or significant life-stage transitions.

These findings first provide a direct empirical response to the central question regarding the eroding predicament of older adults’ peripheral ties. Building on this, they extend the interpretive frameworks of classical aging theories. They also offer a fine-grained, experience-grounded account that enriches the broader literature on digital social ties. Furthermore, they provide valuable references for both age-friendly platform design and for supporting digitally excluded older adults.

## Figures and Tables

**Table 1:** Overview of Participant Groups.

Group	N (M/F)	Age (M ± SD)	Education (Yrs)	WeChat (Yrs)
t1	4 (2/2)	60.75 ± 4.99	15–18+	5–7
t2	4 (2/2)	65.75 ± 3.10	12–18+	8–10
t3	6 (3/3)	62.00 ± 4.24	12–18+	9–13
t4	6 (4/2)	63.25 ± 5.85	9–16	9–13
t5	6 (2/4)	59.25 ± 5.32	12–16	8–12
t6	6 (3/3)	62.75 ± 8.18	9–15	10–13

**Table 2:** Core Focus Group Questions.

No.	Discussion Question
1	Do you use WeChat a lot in your daily life? What do you generally use WeChat for?
2	When did you first start using WeChat? What was the reason you started using it at that time?
3	Do you feel there are any differences between how you use WeChat and how younger people use it, in terms of functions, goals, or expectations for social interaction?
4	Do you use any other social media platforms? Compared to those platforms, what are the social advantages or disadvantages of WeChat?
5	What kinds of people do you have as contacts on your WeChat?
6	How did you add these WeChat contacts?
7	Could you share the specific circumstances of the last two or three WeChat contacts you added recently?
8	What are your thoughts on the usefulness or meaning of these WeChat contacts to you?
9	Since you started using WeChat, could you share a particularly memorable experience you’ve had adding a new contact?
10	How do you usually interact with these contacts on WeChat?
11	Which WeChat features do you typically use for these interactions?
12	Could you share some interaction experiences from the last two or three days?
13	Since you started using WeChat, could you share a particularly memorable interaction experience you’ve had?
14	When you are adding contacts or groups on WeChat, are there any features you find particularly useful? Any that are not useful? Perhaps some features you think should be added or removed?

**Table 3:** Themes, Sub-Themes, and Coding Examples.

Theme	Sub-Theme	Description	Sample Quote (ID)
Typology of Peripheral Ties	Former Acquaintances	Faded ties from pre-retirement life; passively retained on WeChat despite attitudinal disengagement.	“Some classmates were fine back in school, but people change, and it feels awkward to chat on WeChat once the relationship has faded.” (P11)
	Present-day Acquaintances	Contacts tied to current daily life and activities; comprise both individual contacts and group-chat members.	“In these groups, I can see discount information about pharmacies, supermarkets, and department stores; it’s very convenient.” (P22)
	“Net Friends”	Members of interest-based WeChat groups originating from public-account reader communities and QR-code entry from other social media platforms.	“My daughter often calls me a ‘super lurker,’ hiding in ‘all sorts of WeChat groups.’” (P8)
Maintenance Strategies	Existence as Maintenance	Zero-effort passive retention of Former Acquaintances; no interaction, no deletion.	“I actually wanted to delete them long ago, but I was worried it might be awkward.” (P10)
	Information Monitoring with Boundary Management	Reading group messages for instrumental value while suppressing reciprocal engagement and managing privacy.	“I only discuss fishing or other ‘innocuous’ things… saying the wrong thing could cause real trouble.” (P21)
	Strategic Passive Lurking with Network Recomposition	Observation-based engagement with “Net Friends”; replacement of defunct groups to preserve network composition.	“If I’m too active and leave an impression, it would be a bit embarrassing if I stopped talking later.” (P5)
Functional Benefits	Informational Value	Access to non-redundant, heterogeneous knowledge from diverse contacts.	“Many perspectives I hadn’t thought of, which I found very rewarding.” (P9)
	Emotional Value	Belonging, companionship, and emotional resonance through shared interests and vicarious participation.	“Feel like ‘everyone is here with me,’ which makes me feel not so lonely.” (P8)
	Social Role and Identity Support	Sustained sense of family contribution role and retrospective identity liberation.	“My life focus is now entirely on myself.” (P12)

## Data Availability

Our Institutional Ethics Committee requires the protection of participants’ rights and does not allow the raw interview transcripts to be published in full. Data can be made available upon reasonable request.

## Supplementary Materials

The following supporting information can be downloaded at https://www.mdpi.com/article/10.3390/bs16081430/s1. Table S1. Participant Background Information.
