# Supplementary material for: A “Lightweight” Support Circle: How Urban Chinese Older Adults Reconfigure Online Peripheral Ties Through Low-Cost Digital Practices
Source: Behav Sci (Basel). 2026 Aug 19;16(8):1430. doi: 10.3390/bs16081430 (PMC13509251; doi:10.3390/bs16081430)
Supplement: Supplementary file 1 [file behavsci-16-01430-s001.zip › behavsci-4420905-supplementary.pdf]

Supplementary Materials

Table S1. Participant Background Information

ID	Age	Sex	Highest Education Level	Pre-Retirement Occupation/ Industry	Lives with Children (Y/N)	Years of WU	Location
1	56	F	College Diploma	State-Owned Enterprise (SOE) Worker	Y	7	SC
2	64	M	Post-graduate	Middle School Teacher	N	5	SC
3	66	M	Master's Degree (Part-time)	Civil Servant	N	5	EC
4	57	F	College Diploma	Pediatrician	Y	6	EC
5	64	M	Technical School	Private Enterprise Owner	N	8	SWC
6	70	M	Post-graduate	Mid-level Manager, Broadcasting Group	Y	9	EC
7	66	F	College Diploma	Primary School Teacher	Y	10	SC
8	63	F	Postgraduate	University Administrator	N	8	EC
9	67	M	Technical School	TV Station Staff	N	12	SC
10	57	F	Technical School	Tax Bureau Staff	N	10	SC
11	58	F	Post-graduate	Foreign Enterprise Employee	Y	11	EC
12	59	F	College Diploma	State-Owned Enterprise (SOE) Staff	N	9	SWC
13	67	M	High School	Home Decoration Industry	N	13	EC
14	64	M	Bachelor's Degree	Central SOE Staff	N	10	NC
15	61	M	Bachelor's Degree	Vocational College Teacher	N	12	SC
16	65	F	College Diploma	Doctor	Y	10	EC
17	70	M	College Diploma	Forestry Bureau Staff	Y	10	CC
18	66	M	Bachelor's Degree	Engineer	N	9	CC
19	57	M	Junior High School	Construction Engineering Owner	N	13	EC
20	60	F	College Diploma	Foreign Trade Clothing Industry	N	9	CC

21	70	M	College Diploma	Military (Retired)	Y	8	SC
22	61	F	Technical School	Kindergarten Teacher	N	10	SC
23	55	F	Bachelor's Degree	Accountant	N	9	SC
24	58	F	Technical School	Insurance Industry	N	12	SC
25	67	M	Bachelor's Degree	Civil Servant	N	10	EC
26	57	F	College Diploma	Education Bureau Staff	N	10	EC
27	55	F	Technical School	Police Officer	N	9	SC
28	61	M	College Diploma	Primary School Teacher	N	9	EC
29	59	F	Technical School	Women's Federation Staff	Y	10	SC
30	74	M	Junior High School	Private Enterprise Owner	N	12	SWC
31	63	M	College Diploma	Driver	N	10	SWC
32	55	F	Junior High School	Brand Direct Sales Agent	N	13	SWC

Note: Participants are grouped by ID into six focus groups: IDs 1–4 (first group), 5–8 (second group), 9–14 (third group), 15–20 (fourth group), 21–26 (fifth group), and 27–32 (sixth group). In the Sex column, F represents female and M represents male. Educational level corresponds approximately to years of schooling: Junior High School (9 years), Technical/High School (12 years), College Diploma (14–15 years), Bachelor's Degree (16 years), and Postgraduate (18+ years). "Years of WU" denotes years of WeChat use. Location refers to the participant's region of residence. Geographically, China is divided into seven regions: North China (NC), East China (EC), Central China (CC), South China (SC), Southwest China (SWC), Northwest China (NWC), and Northeast China (NEC). To fully protect participants' anonymity and personal interests, variables concerning their economic status and city of residence are not included in this table.
